# All we need to know about internal thoracic artery harvesting and preparation for myocardial revascularization: a systematic review

**DOI:** 10.1186/s13019-021-01733-2

**Published:** 2021-12-27

**Authors:** Matiullah Masroor, Kang Zhou, Chunyang Chen, Xianming Fu, Yuan Zhao

**Affiliations:** 1 Department of Cardiovascular Surgery, The Second Xiangya Hospital of Central South University, 139 Renmin Middle Rd, Changsha, 410011 China; 2 Department of Cardiothoracic and Vascular Surgery, Amiri Medical Complex, Qargha Rd, Afshar, Kabul, Afghanistan

**Keywords:** Internal thoracic artery, Harvesting, Papaverine, Harmonic scalpel, Skeletonization, Pleurotomy

## Abstract

Internal thoracic arteries (ITAs) are the gold standard conduits for coronary revascularization because of their long-term patency and anti-atherosclerotic properties. Harvesting and preparation of ITAs for revascularization is a technically demanding procedure with multiple challenges. Over the last few decades, various methods and techniques for ITAs harvesting have been introduced by different surgeons and applied in clinical practice with different results. Harvesting of ITAs in pedicled or skeletonized fashion, with electrocautery or harmonic scalpel, with open or intact pleura, with clipping the end or keeping it perfused; papaverine delivery with intraluminal injection, perivascular injection, injecting into endothoracic fascia, and papaverine topical spray are the different techniques introduced by the number of researchers. At the same time, access to the ITAs for harvesting has also been studied. Access and harvesting through median sternotomy, mini anterolateral thoracotomy, thoracoscopic, and robotic-assisted harvesting of ITAs are the different techniques used in clinical practice. However, the single standard method for harvesting and preparation of ITAs has yet to be determined. In this review article, we aimed to discuss and analyze all these techniques of harvesting and preparing ITAs with the help of literature to find the best way for ITAs harvesting and preparation for myocardial revascularization.

## Background

As the benefits of arterial grafts over venous grafts both in terms of patency and long-term outcomes has been documented in literature from the last few decades. Surgeons are trying to use more and more arterial conduits for coronary revascularization to achieve these benefits for patients. Internal thoracic arteries (ITAs) are among the most commonly used arterial conduits during coronary artery bypass grafting (CABG). The use of ITAs became the new gold standard after a remarkable study by Loop et al. to know the influence of ITAs on ten years survival and other cardiac events [1]. According to the society of thoracic surgeons (STS) 2016, guidelines on arterial conduits for CABG: The ITAs should be used for left anterior descending (LAD) artery revascularization when indicated with (class of recommendation [COR] I, and the level of evidence [LOE] B). If a second graft is needed with the left internal thoracic artery (LITA), right internal thoracic artery (RITA), or radial artery (RA) should be considered with (COR IIa, LOE B) [2]. The Great Britain and Ireland society of cardiothoracic surgery (2008) database statistics show that 95% of the patients undergoing CABG surgery receive one arterial graft, usually LITA. The STS database statistics for 541,368 patients showed that 92.4 and 4% of patients received LITA and BITA respectively, for coronary revascularization [3]. ITAs have unique molecular mechanisms to combat atherosclerosis formation, and that is why it is considered superior to other grafts for revascularization purposes [4, 5]. Other conduits such as saphenous vein, gastroepiploic artery, splenic artery, radial artery, inferior epigastric artery, and ulnar artery are also recently in practice for coronary revascularization purposes [6].

The harvesting and preparation of ITAs is a broad topic with lots of challenges and controversies. Multiple techniques for harvesting are available, but every method has its pros and cons. These different techniques can influence the result of surgery. These are also the areas of interest with the research still going on.

This article aims to review all the available literature and focus on all these challenges regarding harvesting and preparation of internal thoracic artery for myocardial revascularization. We believe this is the first comprehensive review article to discuss almost all ITAs harvesting and preparation related issues.

## Routes of access to harvest internal thoracic arteries

There are different approaches in the literature and clinical practice on how to access the ITAs for harvesting. In the beginning, chest opening was taking place only through classical sternotomy and ITAs were taken down in pedicle fashion [7]. However, as with advancement in technology and research in cardiovascular surgery, minimal invasive techniques have been introduced. Recently the less invasive procedures for ITAs takedown are: small incision in the anterolateral chest and ITAs takedown under direct vision [8], thoracoscopic ITAs take down [9], and robotic-assisted ITAs take down [10].

### ITAs takedown through classical sternotomy

The traditional method for revascularization was through the median sternotomy. The LIMA was also harvested through the same incision. Surgeons perform sternotomy and harvest LIMA even for single vessels disease (LIMA to LAD) [11, 12]. In this technique, after chest opening through sternotomy, the ITAs retractor is used to elevate the target side, and to have good exposure of the ITA. The ITA is then harvested using electrocautery or harmonic scalpel either in skeletonized or pedicled fashion. Finally, the branches are either clipped and divided with fine scissors or coagulated [13].

Classical sternotomy is the best choice for multiple vessels coronary artery disease to achieve complete revascularization. Because of good exposure and sufficient surgical space, it allows complete harvesting of the ITAs [14]. But this is also the most aggressive and traumatic form of incision to access ITAs. In addition, it may cause post-surgical sternal wound complication such as superficial and deep sternal wound infection, sternal wound dehiscence, and mediastinitis, especially in high-risk patients such as diabetics [15]. Sternotomy may also increase postoperative pain and slow recovery to everyday life compared to other less invasive techniques [16, 17].

### Minimal invasive IMAs takedown under direct vision

The advancement in surgical techniques made it possible to avoid sternotomy for anterior wall single vessel disease revascularization. The less invasive approach for anterior coronary arteries revascularization with ITA (LIMA-LAD) is often performed through anterolateral mini-thoracotomy. This procedure got popular in the last two decades. A small incision in the anterolateral thorax is made in this technique, and access to the thoracic cavity is achieved. A unique retractor is used to elevate the chest wall and gain necessary surgical site exposure, and LIMA is harvested under direct vision. Single lung ventilation is required to achieve maximum surgical field and appropriate exposure. The LIMA is found, harvested, and anastomosed to the target vessel under direct vision [18–21]. Under these circumstances, LIMA harvesting may be incomplete and difficult. The kinking and shortening of the graft for the target revascularized vessel and coronary steal syndrome are the potential complications of this technique [19, 22, 23]. Boonstra et al. harvested the LIMA through anterolateral thoracotomy under direct vision and explained the procedure in detail. They harvested the LIMA in pedicled fashion in 20 patients from February to June 1996 [24]. Lung disease with low vital capacity or forced expiratory volume 1, pleural adhesions, limited surgical space such as in obese patients, and chest deformities are contraindications for this procedure [25].

### Thoracoscopic IMAs takedown

In this procedure, the IMAs are harvested with the help of a manually adjusted thoracoscope. Nataf et al. published the results of a one-year study in which LIMA of 32 patients was harvested with a thoracoscope. The procedure they described was to put the patient in hemi oblique position. Single lung ventilation with a double lumen ET tube was achieved to collapse the left lung and get enough surgical space. Three trocars were inserted with a small 15 mm thoracic incision at the 4th, 6th ICS at the midaxillary line and in the 5th ICS at the anterior axillary line. The surgeon was standing on the left side of the patient. Usually, a thoracoscope was inserted through the 5th ICS and instruments through the other two ports. A rigid 10–30 degree thoracoscope was used for visualization of the thoracic cavity and identification of IMA. The IMA was usually identified very easily in its first part, just distal to its origin from the subclavian artery where only a thin layer of parietal pleura covers it. Fats cover the middle part, and the distal part is intramuscular usually. The IMA was harvested from proximal to distal after the incision of the parietal pleura with diathermy. The collaterals were either clipped or coagulated with diathermy. The distal end was trimmed between two clips after general heparinization. They believe this technique allows complete harvesting of left IMA from the subclavian artery to the 6th intercostal branch. After harvesting, the incision in the 5th ICS was extended up to 4 cm, and a mini-thoracotomy was performed to anastomosed the already harvested IMA to the LAD. The thoracotomy incision did not need retraction because the IMA was already harvested. They also believe skeletonized harvesting was easier than pedicled harvesting together with fascia and muscle. During skeletonization, collaterals could be well visualized and coagulated or clipped far from graft to avoid harming the graft. The collateral division was effective through diathermy by direct coagulation rather than by clipping. It was more practical, rapid, and avoided transferring instruments repeatedly through the same trocar. Their study showed no conversion to sternotomy because of the LIMA damage and no reoperation for bleeding. The average LIMA harvesting time was 58.7 min ranging from 20 to 130 min [22]. Benetti et al. also performed this procedure almost the same way in 30 patients, but they harvested LIMA in pedicle fashion. In their experience, patients with this procedure may be extubated in OR, and some patients left the OR by walking [26]. Robin et al. claimed that in their three-year experience of thoracoscopic IMA harvesting, they could completely harvest the ITA, avoid kinking of the graft, coronary steal syndrome, and shortening of the graft; the problems which are considered to be related to the direct vision IMAs harvesting [27].

### Robotic assisted IMAs takedown

Robotic assisted cardiac surgeries' technology is the most advanced technology introduced in cardiac surgery. Robotic assisted ITAs takedown is the primary step for TECAB surgeries. Robotic assisted ITAs takedown is also the least invasive procedure performed for ITAs harvesting and coronary revascularization. Davinci robotic system is commonly used for this purpose. The IMAs are taken down with the help of robot, and anastomosis occurs either through small anterolateral thoracotomy under direct vision or through robot assistance (TECAB). Oehlinger et al. published their experience after 100 cases in which LIMA was harvested with the help of a robot (da Vinci surgical system). They introduced the technique to put the patient in a 30 degree right lateral position with single lung ventilation. The camera port was inserted at the anterior axillary line at the 5th ICS. After 8–10 mmHg pressure of CO2 insufflation, instruments ports were inserted under thoracoscopic vision at 3rd and 7th ICS at the mid axillary line. The ITA was exposed and harvested from 1st to 5th ICS with the help of electrocautery, which was fixed at 20 W. The branches were clipped with endoscopic clips. After heparinization, the distal end was partially incised to check the flow. After free flow was observed, the distal end of the graft was prepared for anastomosis. Four cases of the LIMA injury, three (6%) in the first half and one (2%) in the second half of the study, occurred. One patient converted to sternotomy because of the LIMA damage, which affected the flow. In one patient, the damage caused by electrocautery ended up with end-to-end anastomosis of the LIMA. For the other two cases, intraoperative angiography was necessary, which showed stenosis in one patient probably because of electrocautery, and intramural hematoma in other case compromised blood flow. All cases left the operating room with patent LIMA. Robotic assisted ITA harvesting was associated with a significant learning curve. The median time for LIMA harvesting in their study was 48 min ranging from 19 to 120 min. The harvesting time decreased to 34 min (median time) in the last 10 cases from 140 min (median time) in the first 10 cases. The harvesting time decreased to an acceptable duration of 30–60 min after 20 surgeries and further decreased to less than 40 min after 70 cases. LIMA harvesting time was neither influenced by demographic factors nor variation in thoracic dimensions. They concluded that IMA harvesting could be done safely and with an acceptable duration after passing through the learning curve [28]. Robin et al. discussed the benefits and feasibility of videos assisted robotic bilateral internal thoracic artery harvesting using the voice control AESOP 2000 robot system. They believe this new minimally invasive procedure was more precise, tremor free, and faster than conventional thoracoscopic BITA harvesting. Positioning the arm on the right side at the right hip level allowed harvesting of both ITAs without changing the initial position of the arm. Harvesting was efficient by saving the picture and position of the arm in the robot memory between the steps for an abrupt return to the previous position. It kept the arm in the same position and immobile, while changing position at different surgery steps to avoid multiple contacts between thoracic organs and thoracoscope [27]. Ishikawa et al. also performed a study of 10 patients and skeletonized LIMA with a da Vinci device. In all cases, the LIMA was successfully harvested. The harvesting time was 38 ± 25.2 min. The length of the graft on average was 16.2 ± 3.1 cm. The camera port in their study was inserted in the 4th ICS at the anterior axillary line. After CO2 insufflation of 6–12 mmHg, the two other instruments ports were inserted at the 2nd ICS at the anterior axillary line and in the 6th ICS at the midaxillary line. One patient experienced hypotension with systolic BP of less than 50 mmHg. In this patient, the camera port was more lateral to the anterior axillary line, and the pressure of CO2 was increased to 18 mmHg. After de sufflation, the patient was stable and the procedure continued with CO2 pressure of 14 mmHg [29]. Fujita et al. shared their initial experience with robotic LIMA takedown. Out of 33 patients, LITA was successfully harvested in 30 patients (91%), while three patients (9%) needed conversion to sternotomy because of bleeding. The risk of damage to the LIMA was associated with age (*p* = 0.0012). The average harvesting time was 68 min. They concluded that off-pump MIDCAB combine with robotic LIMA harvesting is less invasive than the standard procedures. The difficulty includes controlling bleeding from the graft, especially in the elderly, and identifying the target revascularized vessel [30]. A study by Merwe et al. discussed the reason for conversion to sternotomy in robotic enhanced coronary revascularization. Their study included 759 RE-MIDCAB patients, out of which 30 patients (4%) needed conversion to sternotomy. The reasons for conversion were lung adhesion 11 (36.7%), failure to maintain single lung ventilation 1 (3.3%), graft shortening 3 (10.0%), graft damage 5 (16.7%), spasm or no flow 3 (10.0%), and poor visualization of the target vessel 3 (10.0%). They advised technical skills development, careful planning, and team approach under a surgeon's leadership to avoid conversion to sternotomy [31].

## Hydro dissection technique for ITAs harvesting

Skeletonization of ITAs has shown its superiority in providing extra length to the graft and avoiding sternal wound complication, which will be discussed in the next section. It also decreases the amount of trauma to the chest wall. But it is technically challenging and difficult to perform compared to pedicled harvesting, especially for less experienced surgeons. There is a possible risk of ITAs damage during skeletonized harvesting. To avoid the risk of injury and make the harvesting easier, Saxena et al. introduced a safe and simple method of hydro dissection, in which 10 to 20 ml of normal saline was injected into the endothoracic fascia to create a plane of dissection. After the chest opening, they opened the pleura widely and injected saline in the proximal or middle portion along the course where ITA was visible with the help of a 23G needle and 20 ml syringe. The injection was made a few mm away from the graft to avoid damaging the artery or vein. Injection of normal saline anterior to endothoracic fascia raises the fascia along the whole length of the parasternal area. Normal saline not only enters the plane between ITA and its bed, making a thin cushion of fluid along with the graft and making harvesting easier, but also allows saline to enter between the ITA and chest wall. At the same time, it can decrease the conduction of heat created by the diathermy. An opening was made and extended along the graft. Once the endothoracic fascia was opened, the tissues do not look very edematous, and it was not difficult to work through the thin layer of normal saline. The harvesting time, according to their study, were 20 min. They believe the technique was safe, easy, and did not require any special instruments. Harvesting of LITA in their study was without any complication of injury or dissection. The histopathology examination of the distal end also confirmed the intact integrity of the graft [32]. Bahcivan et al. injected papaverine instead of normal saline and found that papaverine helps decrease harvesting time and avoid spasm and reuse of papaverine after harvesting [33]. Saxena et al. also used papaverine for harvesting purpose. They believe papaverine can develop a plane for ITAs dissection, but they encountered bleeding from side branches and accompanying vein because of the vasodilatory effect [34].

## Skeletonized versus pedicled ITAs harvesting

As the conventional method of harvesting ITAs was pedicled harvesting [7], studies showed that pedicled harvesting could affect the sternal blood flow, especially in bilateral ITAs harvesting, leading to sternal wound complication [35]. To avoid sternal wound complications, surgeons tried to harvest the ITAs in skeletonized fashion, which has improved the outcomes of these surgeries. Rubens et al. compared skeletonized bilateral internal thoracic artery (BITA) harvesting with non-skeletonized BITA,  and concluded that skeletonization plays a protective role in avoiding sternal wound infection (SWI). Even though the skeletonized BITA group in their study had many risk factors compared to the non-skeletonized group, there was still a significant effect of skeletonization on decreasing sternal wound incidence [36]. A sub study of an arterial revascularization trial (ART) relating harvesting techniques with sternal wound infection found that skeletonized BITA grafting having the same risk of sternal wound complication as standard pedicled SITA grafting, whilst the skeletonized SITA grafting did not add any further benefit compare to pedicled SITA harvesting [37]. A systematic review and meta-analysis also compared the effect of BITA skeletonized harvesting. The overall odds ratio (OR) of sternal wound infection showed a statistically significant difference favoring skeletonization. Still, in sensitivity analysis, the statistical difference favoring skeletonization was limited to patients with diabetes mellites [38]. Another systematic review and meta-analysis by Hu et al. reviewed the literature from 1966 to 2010 for control trials and found 22 eligible trials comparing skeletonized versus pedicled ITAs harvesting techniques. They found skeletonization offer a significant increase in length, caliber, and flow capacity of graft compared to pedicled harvesting on angiographic results at midterm outcomes. Skeletonization was also associated with low SWI and lower thoracic wall pain. They concluded that when BITA is used for revascularization purpose, the skeletonization reduces the SWI compared to pedicled harvesting. It also decreased blood loss and intubation time. Most importantly, skeletonization improved prognostic benefits and reduced adverse cardiac events in high-risk patients [39]. Choi et al. studied the effect of skeletonization on free blood flow compared to pedicled harvesting. They found that skeletonization can avoid an early decrease of blood flow and spasm of the graft even without the use of papaverine. Their study proved that skeletonized graft flow is as efficient as the flow of pedicled graft with intraluminal papaverine injection [40].

Mazur et al. in an RCT for skeletonized versus pedicled LIMA harvesting found that skeletonization of LIMA is associated with lower postoperative mediastinal drainage. On 24 h observation, mediastinal drainage of skeletonized LIMA harvesting group was lower by 26% compared to pedicled harvesting. The pedicled harvesting group also received more fresh frozen plasma unit compare to the skeletonized group, even though the coagulation profile of both groups was similar [41]. On the other hand, Lazar et al. reviewed the literature of 50 years and concluded that DSWI is a multifactorial process and is independent of skeletonization techniques. Therefore, the skeletonization of ITAs alone is not enough to avoid SWI [42]. As skeletonization needs some expertise and is time-consuming; another simplest method, semi skeletonization which is as simple as pedicled harvesting technique was discussed by Opas et al. in an RCT and concluded that semi skeletonization harvesting provides greater graft length and intraoperative diastolic flow of the graft compared to pedicled harvesting graft [43].

Even though skeletonization decreases the risk of deep sternal wound infection (DSWI) and other sternal wound complication, some researchers believe that skeletonization can harm the graft and affect the graft's long-term patency. Dreifaldt et al. designed an RCT to assess if skeletonization can jeopardize the patency of ITAs graft? They followed the patients at three and eight years interval with angiography and CTA respectively. They concluded that skeletonization of ITA could be performed without the fear of affecting the patency of ITA graft. According to their study, the main factor of graft failure was not the method of skeletonization but target graft stenosis less than 70% [44]. A systematic review and meta-analysis compared graft patency of skeletonized versus pedicle harvesting techniques, and found no statistically significant difference for graft occlusion in overall OR in both groups. They also found no difference in left and right ITAs in the sensitivity analysis. They also did not find a statistically significant coefficient for graft occlusion and proportion of age, female, diabetes mellitus, renal failure, urgent surgery, and off-pump surgeries in meta-regression. They concluded that skeletonized ITAs harvesting was not inferior to pedicled ITAs harvesting [45].

Another randomized comparative study by Puslecki et al. compared endothelial integrity of skeletonized versus pedicled harvesting of ITAs grafts using the histological and immunohistochemical examination. They randomized 120 patients, 60 to each group skeletonized and pedicled. They observed a segment of ITA histologically under the light microscope and also evaluated the endothelial expression of CD31, CD34, CD133, and nitric oxide synthase immunohistochemically. In both groups' LITA segment under the light microscope, there was no significant arterial wall damage such as dissection, disruption, sub adventitial hematoma, or thrombosis. Furthermore, on the immunohistochemical evaluation of proteins expression, there was no difference in endothelial expression of CD34, CD133 antigens, and nitric oxide synthase in both groups, which showed the same regeneration potential of the grafts' functional integrity. At the same time, CD31, which is the marker of the endothelium's morphological integrity, was stronger in the pedicled group [46].

## Harmonic scalpel versus electrocautery harvesting

The conventional choice for skeletonization and harvesting of the ITAs is electrocautery (EC), but as the surgeons experienced some degree of difficulties with EC, especially in minimally invasive procedures, where the surgical field is limited, and more smoke production is troublesome by blurring surgical filed which need continuous suction, a new technology of harmonic scalpel (HS) which use ultrasonic energy, produce less smoke, avoid heat-induced injury, and is beneficial for both patient and surgeon has been developed. Urso et al. in a randomized comparison of harvesting methods compared EC and HS and found that the intraoperative mean flow is similar in both groups and is independent of energy source. They used a transient time flowmeter (TTF) to measure the flow at three different times, before harvesting the IMA, after harvesting was completed, and after CPB in the anastomosed graft in both groups. There was no statistically significant difference at any stage of flow evaluation in the two groups. The mean flow at time two (after harvesting) was decreased in both groups, which can be attributed to vasospasm. Still, the flow at time three (after CPB) was more than time one (beginning of harvesting) which is probably by the reversibility of spasm and independent of energy source [47]. Kieser et al. found that HS is faster yet safe compared to conventional skeletonization techniques for ITAs. After the learning curve, the harvesting time with HS was half of the traditional harvesting techniques in which a cautery tip was used as a dissector. HS rarely damaged the ITAs and had comparable major adverse events related to ITAs used, compared with conventional harvesting techniques. They also believe approximately ten harvestings of ITAs are needed for those already familiar with skeletonization techniques and twenty harvestings for those not familiar with skeletonization techniques to reach a comfort level with HS skeletonization [48].

HS produces less smoke, generates less heat, needs fewer surgical clips, and can reduce the frequency of instruments transfer because it cuts, coagulates, and splits all tissues compared to regular EC. Therefore, it is a better alternative and can decrease surgery time [48–50]. Kiaii et al. also used HS harvesting 100 patients' ITAs and found that HS greatly facilitate IMAs harvesting [13]. Higami et al. evaluated the branches of ITAs divided with HS histologically and physiologically. They divided the patients into three groups based on the distance of the branch divided with HS from its origin, i.e. 0, 1, and 2 mm. In group one, 8 out of 15 branches showed discontinuity of vessel wall probably because of insufficient sealing, but in other two groups continuity of the vessel wall was confirmed. The ITA itself in group one had tissue damage which was absent in group two and three. On physiological evaluation 2 out of 24 branches burst under the pressure of 350 mmHg while 22 resists up to 350 mmHg pressure. Both burst branches were less than 1 mm in length 0.3 and 0.5 respectively. The duration of cutting and coagulation was also shorter for these two branches compared to other 22 branches. They concluded that HS is a reliable and safe method if branches are divided at least 1 mm far from the origin with a sufficiently slow speed [51]. The temperature in the surrounding tissue is less than 80 °C when HS is used, while it is more than 300 °C when electrocautery is used. Thus, the use of a Harmonic scalpel compared to electrocautery has a much lower temperature which has direct effect in avoiding thermal induce injuries of ITAs and surrounding tissues [52]. Orejola et al. also compared HS with EC and found that HS is effective and safe for ITAs harvesting after comparable histological, hemodynamic and clinical outcomes [53]. Pektok et al. studied the effect of HS and EC on postoperative sternal perfusion in patients who underwent LITA harvesting in a pedicled fashion. They found that HS does not offer any beneficial effect regarding sternal blood flow [54].

## Open versus closed pleural ITAs takedown

Research has also been done on ITAs takedown with and without intact pleural integrity. Usually, the pleurotomy is done during harvesting to provide better exposure to the artery and take the ITA down in a pedicled fashion. Still, some researcher believes it may have an adverse effect on pleural function. Rezk et al. performed a study comparing both groups (ITA harvesting with pleurotomy and extrapleural ITA take down) by assigning 50 patients to each group. They performed pre and postoperative spirometry and also recorded postoperative pulmonary complication in both groups. They found that the forced expiratory volume 1 (FEV1%), Forced vital capacity (FVC%), and FEV1/FVC ratio in the closed pleural group was significantly improved compared to the open pleural group on the fifth postoperative day, at the time of discharge, and on 30th postoperative day. In addition, there was lower postoperative pleural complication such as atelectasis and pleural effusion in preserved pleural integrity group compared to the pleurotomy group. They concluded that preservation of pleural integrity during ITAs harvesting has a beneficial effect on postoperative lung function and having lower pulmonary associated complications [55]. Many other studies have also shown the beneficial effects of extrapleural ITAs takedown compared to pleurotomy ITAs takedown. Better pulmonary function, lower pulmonary complications (pleural effusion, atelectasis), less postoperative bleeding and pain, low thoracic wall complications, less hospital stay, and less cost are the beneficial effects of extrapleural IMAs take down [56–63]. Usually, the extrapleural ITAs takedown will be performed in skeletonized while pleurotomy ITAs takedown in pedicled fashion. The benefits of pulmonary function may not be attributed to skeletonization. Bonacchi et al. conducted a randomized study and divided the patients into three groups. Pedicled ITA harvesting with pleurotomy, skeletonized ITA harvesting with intact pleura, and skeletonized ITA harvesting with pleurotomy. Skeletonized ITA harvesting with intact pleura group showed better pulmonary function outcomes than skeletonized pleurotomy group, which showed the benefits of pulmonary function are attributed to intact pleural integrity itself and not skeletonization procedure [56].

## Traditional (clipped) versus modified (nonclipped) ITAs preparation

The traditional way of harvesting was to prepare the ITAs in pedicled form, clipped and cut the end of the graft and covered the graft with papaverine soaked gauze until anastomosis. The modified technique is to harvest the ITAs and keep it in situ connected to systemic circulation without clipping the end until anastomosis. Some researchers believe that the clipping approach has adverse effects on graft quality. Buyukates and colleagues, performed an immunohistochemistry study to know the histological difference of endothelial integrity and nitric oxide synthase amount secreted by endothelial cells. The segment of the artery received from the bifurcation area was observed after immunohistochemical methods. The tunica media of the clipped artery was thinner than the nonclipped artery group, which may be attributed to the high luminal pressure in the clipped artery group. In addition, the immunostaining was absent in the striped regions of luminal endothelium of the clipped artery. In contrast, a noticeable amount of immunostaining occurred in the luminal endothelium of the nonclipped artery group. So, it was concluded that the clipped artery technique jeopardizes the integrity of endothelium and decreases the amount of nitric oxide production [5].

Grapow and colleagues investigated the biomarkers for endothelial dysfunction with the help of ELISA and structural changes in the endothelial layer of the artery under the electron microscope. They found that the soluble thrombomodulin and human soluble platelet selectin levels were significantly higher in clipped groups than perfused groups. There were also profound endothelial lining changes in the clipped group, while no signs of endothelial cell loss found in the perfused group under the electron microscope. So, their biochemical and electron microscopy results confirmed that perfused ITAs maintain the functional integrity of the endothelium while the functional integrity in the clipped artery is disrupted [64]. Another study performed a different research methodology to know the effect of clipping on ITAs' endothelium. They used contractile substances (endothelin-1, noradrenaline, 5-hydroxytryptamine, and potassium chloride) and relaxant substances (acetylcholine and sodium nitroprusside) on the sections of the artery. They concluded that occluding the end of ITA even for a short period may disrupt the endothelial function. On the other hand, keeping the artery perfused has the endothelial preservation effect, which may contribute to short and long-term graft patency [65].

## Different methods of papaverine delivery

As the harvesting of ITAs is completed, different surgeons will perform various procedures to keep the ITAs in good shape until anastomosis to the target vessel. As discussed above: it either be kept in situ; or cut to serve as a free graft until anastomosis. ITAs have their intimal layer, which contains the muscles same as all other arteries. So, there is the potential of spasm during harvesting, after harvesting, and in the postoperative period. ITAs spasm has been documented to occur in an early postoperative period which is lethal and need immediate and aggressive intervention for survival. Sarabu et al. recommended assessment of postoperative angina and marked ECG changes with coronary angiography. They suggested intracoronary nitroglycerine infusion or sublingual administration of nifedipine to be used for treatment as necessary. They also recommended reoperation and evaluation of graft for immediate postoperative, acute, intractable hemodynamic collapse [66]. To deal with this issue, studies have done and shown the positive effect of papaverine for relieving spasm, and this drug has been widely used for this purpose [67, 68]. Other vasodilators such as sodium nitroprusside and milrinone have also been effective in relieving spasm [69–71]. Multiple techniques for papaverine delivery have been studied, such as intraluminal papaverine injection, perivascular papaverine injection, submerging of the ITAs graft in papaverine solution, papaverine injection in the endothoracic fascia, and topical papaverine spray. Studies have shown that intraluminal papaverine administration offers more benefits than other techniques, but it is associated with the risk of intimal injury [33, 68, 72, 73]. A study compared the effect of papaverine administration by two different techniques, topical spray, and perivascular injection while administering the same dose to both groups. The systemic blood pressure was kept at 70 mmHg for both groups during measurement. They measured the LIMA graft per minute flow before administering papaverine which was not statically different 51.9 and 55.1 ml/m between the groups. They measured the flow again 20 min after papaverine administration and found that graft flow in the perivascular injection group increased with statically significant difference 87.2 and 104.7 ml/m [74]. To avoid mechanical injury to the intima of ITAs, some researcher believes that perivascular injection of papaverine to the periarterial tissue of the pedicle is a safe and effective alternative to intraluminal and topical papaverine application [68, 75]. This approach is also applicable when a nonclipped artery technique is used where intraluminal delivery would not be possible because the IMAs would not have been trimmed and would still be connected to the systemic circulation.

Another approach for papaverine injection to the skeletonized graft harvested without the surrounding tissues is injecting the papaverine into endothoracic fascia before harvesting. Bahcivan et al. studied this approach by comparing papaverine delivery through three different methods, injecting into endothoracic fascia, periarterial tissues of pedicle graft, and intraluminal injection and measured free flow at two different times. They found that mean blood flow in group one was much better than the other two after harvesting, and mean flow was almost similar in all groups before anastomosis. They concluded that endothoracic fascia papaverine injection is a reliable method and decreasing ITAs harvesting time without any damage and spasm [33]. Sasson et al. designed a study comparing different vasodilators effect on IMAs, which were used topically. They divided the patients into five groups. Each drug: normal saline, papaverine, nitroglycerine, and sodium nitroprusside, were used topically in 4 groups, and the combination of papaverine and normal saline was injected in periarterial tissue in the 5th group. They harvested the IMAs in a wide pedicle greater than 2 cm fashion, and cut the graft at least 3 cm proximal to the bifurcation. They measured the graft flow twice: once after trimming the IMA; and other time after using vasodilators and before cardiopulmonary bypass. They noticed significant increase from flow 1 to flow 2 in all groups but there was no difference in the increase of flow among the groups from flow 1 to flow 2. They concluded that if the IMAs are harvested in the above-mentioned fashion, and early graft flow is greater than 40 ml/m, no topical vasodilators are needed [76].

## Conclusion

After studying and analyzing all the available literature, we concluded that the best way to harvest the ITAs is in a skeletonized fashion, with intact pleural integrity, and with the help of a harmonic scalpel. Though it is technically demanding, it decreases the risk of complication. Harvesting with intact pleura can have a beneficial effect on lungs function after surgery. The harmonic scalpel is a safe and effective alternative to electrocautery. It can help decrease surgical duration because it can perform multiple tasks and avoid frequent transfer of instruments in endoscopic surgeries. It is better to keep the ITA perfuse without trimming and clipping the end until anastomosis to prevent endothelial damage. The best way of papaverine delivery is perivascular injection in pedicled graft as it avoids the risk of endothelial injury and has more benefits than topical spray. For skeletonized grafts, papaverine can either be injected into endothoracic fascia before harvesting, or topically sprayed and the ITA cover with gauze soaked with papaverine. The least invasive method to access ITAs for harvesting is endoscopic assisted harvesting with either robot or manually manipulated thoracoscope followed by harvesting through anterolateral thoracotomy under direct vision and harvesting through median sternotomy.

## Data Availability

All data generated or analyzed during the current study are included in this published article.

## Abbreviations

ITAs: Internal thoracic artery
LITA: Left internal thoracic artery
LAD: Left anterior descending artery
CABG: Coronary artery bypass grafting
MIDCAB: Minimal invasive direct coronary artery bypass
TECAB: Total endoscopic coronary artery bypass
TTF: Transient time flow meter
EC: Electrocautery
HS: Harmonic scalpel
